# Valganciclovir Inhibits Human Adenovirus Replication and Pathology in Permissive Immunosuppressed Female and Male Syrian Hamsters

**DOI:** 10.3390/v7031409

**Published:** 2015-03-23

**Authors:** Karoly Toth, Baoling Ying, Ann E. Tollefson, Jacqueline F. Spencer, Lata Balakrishnan, John E. Sagartz, Robert Mark L. Buller, William S. M. Wold

**Affiliations:** 1 Department of Molecular Microbiology and Immunology, Saint Louis University School of Medicine, 1100 S. Grand Blvd., St. Louis, MO 63104, USA; E-Mails: yingb@slu.edu (B.Y.); tollefae@slu.edu (A.E.T.); spencejf@slu.edu (J.F.S.); bullerrm@slu.edu (R.M.L.B.); woldws@slu.edu (W.S.M.W.); 2 Department of Biology, Indiana University Purdue University Indianapolis, Indianapolis, IN 46202, USA; E-Mail: latabala@iupui.edu; 3 Department of Comparative Medicine, Saint Louis University School of Medicine, 1100 S. Grand Blvd., St. Louis, MO 63104, USA; E-Mail: jsagartz@7thwavelabs.com

**Keywords:** adenovirus, antiviral, hamster, valganciclovir

## Abstract

Adenovirus infections of immunocompromised pediatric hematopoietic stem cell transplant patients can develop into serious and often deadly multi-organ disease. There are no drugs approved for adenovirus infections. Cidofovir (an analog of 2-deoxycytidine monophosphate) is used at times but it can be nephrotoxic and its efficacy has not been proven in clinical trials. Brincidofovir, a promising lipid-linked derivative of cidofovir, is in clinical trials. Ganciclovir, an analog of 2-deoxyguanosine, has been employed occasionally but with unknown efficacy in the clinic. In this study, we evaluated valganciclovir against disseminated adenovirus type 5 (Ad5) infection in our permissive immunosuppressed Syrian hamster model. We administered valganciclovir prophylactically, beginning 12 h pre-infection or therapeutically starting at Day 1, 2, 3, or 4 post-infection. Valganciclovir significantly increased survival, reduced viral replication in the liver, and mitigated the pathology associated with Ad5 infection. In cultured cells, valganciclovir inhibited Ad5 DNA replication and blocked the transition from early to late stage of infection. Valganciclovir directly inhibited Ad5 DNA polymerase *in vitro*, which may explain, at least in part, its mechanism of action. Ganciclovir and valganciclovir are approved to treat infections by certain herpesviruses. Our results support the use of valganciclovir to treat disseminated adenovirus infections in immunosuppressed patients.

## 1. Introduction

There are close to 70 types of human adenoviruses (Ads) described in the literature, named Ad1, Ad2, *etc.*, that form seven species (A–G). Many Ad types are ubiquitous and quite benign, but under certain circumstances Ads can cause serious infections of the respiratory and gastrointestinal tracts, the eye, and other organs, and Ads can be very dangerous in immunocompromised patients (reviewed in [1,2]). There are no drugs approved specifically to treat Ad infections, *i.e.*, no controlled clinical trials have been completed leading to approval of any anti-Ad drug.

Cidofovir (CDV), an acyclic nucleoside phosphonate analog of 2-deoxycytidine monophosphate, is often used in the clinic for immunosuppressed pediatric allogeneic hematopoietic stem cell transplant patients with rising titers of Ad in the blood as determined by quantitative polymerase chain reaction (qPCR) [1,3,4,5]. Depending on the study, from 3% to 47% of such patients suffer disseminated Ad infections, and mortality can approach 100%. CDV (a monophosphate) is phosphorylated to the diphosphate form by cellular kinases; the diphosphate form is used as a substrate by the viral DNA polymerase, leading to incorporation of CDV and, eventually, to chain termination [6]. The specificity of CDV for many DNA viruses comes from the higher affinity of the viral enzymes for CDV-diphosphate. Unfortunately, CDV poses risk of kidney toxicity.

A newly developed compound, brincidofovir (BCV) (formally named CMX001), is being evaluated in clinical trials [7] and case studies [8] with promising results. However, pivotal clinical trials must be completed before BCV can be approved. BCV is a lipid-linked derivative of CDV that is much more bioavailable than CDV and less nephrotoxic. BCV can diffuse easily through cellular membranes, after which the lipid moiety is removed by phospholipase C, leaving the CDV inside the cell.

Recently, we have characterized CDV, BCV, and ribavirin regarding their anti-Ad efficacy in the immunosuppressed Syrian hamster model [9]. This animal model was developed in our laboratory [10,11]. In this model, Syrian hamsters are immunosuppressed by treatment with cyclophosphamide (CP), an agent that reduces white blood cell counts by ~7 fold in a few days [10,12,13,14]. While immunocompetent Syrian hamsters are permissive for species C human Ads [15,16], immunosuppression accentuates the pathology caused by the virus because the immune system cannot eliminate the infection [10]. Ad5 administered intravenously (i.v.) is able to cause pathology and to replicate to high levels for extended periods in the liver and other organs. We found that CDV and BCV were very efficacious against human Ad5 in this animal model, while ribavirin was largely ineffective.

In addition to CDV, BCV, and ribavirin, another small molecule inhibitor that has been used occasionally in the clinic against Ad, mostly in case studies, is ganciclovir (GCV) (reviewed in [1,3,5,17,18,19,20], an analog of 2-deoxyguanosine. GCV is approved to treat retinitis caused by human cytomegalovirus (HCMV) (which can lead to blindness) and it is used for emergent HCMV infections in immunosuppressed transplant patients and AIDS patients. GCV must be phosphorylated to GCV-monophosphate by an HCMV-coded protein kinase (the UL97 protein kinase). Cellular kinases generate the GCV-diphosphate and GCV-triphosphate, the latter being a substrate for the viral DNA polymerase. The specificity for virus-infected cells comes from the initial phosphorylation of GCV by the viral kinase (cellular kinases phosphorylate GCV poorly if at all) and by more efficient use of GCV-triphosphate by the viral DNA polymerase than by cellular DNA polymerases. Unfortunately, there are very limited clinical data on the efficacy of GCV against Ad (see Discussion). One might think that GCV would not be active against Ad, considering, by analogy with HCMV, that GCV must be phosphorylated initially be a viral kinase, and that Ad is not known to encode such a kinase. Nevertheless, when we evaluated GCV in the hamster model, we found that its anti-Ad activity was similar to that of BCV [21].

Presently, valganciclovir (VGCV), a valyl-ester prodrug of GCV, is the most frequently used drug to prevent CMV infection after solid organ transplantation [22]. VGCV has much better oral availability than GCV, reaching 60% [23]. After uptake, the valine moiety is cleaved by intestinal and hepatic cellular esterases, yielding GCV, which, as mentioned, must be phosphorylated into its monophosphate form by a viral kinase and then into its triphosphate form by cellular kinases for its anti-herpesvirus activity. VGCV is approved for the treatment of HCMV retinitis associated with AIDS as well as disseminated infections in patients immunosuppressed during transplantation, especially of heart, kidney, and kidney-pancreas.

We are unaware of any reports in the clinical literature or in animal studies on the use of VGCV to treat disseminated Ad infections. To determine if VGCV had anti-Ad activity, we tested if the drug could mitigate the effects of intravenous challenge with Ad5 in immunosuppressed hamsters. We report here that VGCV is very effective against Ad5-induced pathology and replication in the liver, even when the drug is given 4 days post-infection (p.i.) to hamsters challenged with the LD_50_ of Ad5, and 2 days p.i. when the hamsters are challenged with the LD_90_ of Ad5. At 2–4 days p.i., the titers of Ad5 in the liver are extremely high (~10^10^ infectious units per gram of liver) in untreated hamsters. Thus, it is especially promising that VGCV is efficacious at this stage of infection. Further, we provide initial evidence that VGCV (or GCV) may exert its anti-Ad5 effect by novel mechanisms—by directly inhibiting the Ad5 DNA polymerase and disturbing the intracellular nucleotide balance.

## 2. Materials and Methods

### 2.1. Materials

A wild-type human Ad5 isolate, named Ad5 *wt500*, was used in all animal experiments. Ad5 *wt500* was derived by plaque purification from an Ad5 stock purchased from ATCC in our laboratory. The genome sequence of the isolate was verified to match the wild-type consensus sequence. The titer of the viruses was determined by plaque assay on human A549 cells. VGCV (batch 20120410) was purchased from 2A Pharmachem (Lisle, IL, USA), and dissolved in water at 3, 10, or 20 mg/mL for the animal doses of 30, 100, or 200 mg/kg. Syrian hamsters (*Mesocricetus auratus*) of approximately 100 g weight were purchased from Harlan Laboratories (Indianapolis, IN, USA).

### 2.2. Syrian Hamster Experiments

All hamsters were immunosuppressed using CP. CP was administered intraperitoneally (i.p.) at a dose of 140 mg/kg, and then twice weekly at a dose of 100 mg/kg for the remainder of the experiment.

For the studies in which the drug was dosed therapeutically, the animals were distributed into groups of 15 hamsters each, immunosuppressed with CP, and then injected i.v. with vehicle or 1.2 × 10^10^ PFU of Ad5 per 100 g body weight. We administered VGCV by oral gavage at 200 mg/kg, starting at 12 h before, or 1 day, 2 days, 3 days, or 4 days after Ad5 injection. In the Results and Figure Legends, these groups are referenced as −12 h, D +1, D +2, *etc.* After the initial administration of VGCV, it was administered twice daily for the remainder of the study. An Ad5-infected group that did not receive drug, and groups that received virus vehicle and drug vehicle only or virus vehicle plus drug (started at 12 h before challenge) were used as controls.

For all animal experiments, the body weights and any signs of morbidity of the animals were recorded daily. On day 5 (for the experiment with female hamsters) or 7 (for the experiment with male hamsters), five hamsters of each group (designated at the start of the experiment) were sacrificed, and gross pathological observation was performed. Serum and liver samples were collected. Liver was fixed in 10% neutral-buffered formalin and tissues were subsequently routinely processed for paraffin embedding, sectioned, and stained with hematoxylin and eosin prior to microscopic evaluation. Virus burden in liver was determined by tissue culture infectious dose 50% (TCID_50_) assay. The serum was analyzed for liver transaminase levels. The remaining 10 hamsters were sacrificed at 16 (for the experiment with female hamsters) or 13 (for the experiment with male hamsters) days post challenge. Hamsters that became moribund before day 16 were sacrificed as needed and processed as described above. Thus, there are two endpoints for these studies. One, collected from 10 animals, is survival and body weight gain/loss. The other, collected from 5 animals for the day 5 time point, is virus burden in the liver and serum transaminase levels.

The severity of microscopic pathology was scored on a scale ranging from 0 to 4 (no lesions: 0, minimal: 1, mild: 2, moderate: 3, marked: 4). Each group was assigned a composite severity score by averaging the individual scores. The distribution of the lesions was described as focal, multifocal, or diffuse.

### 2.3. Determining the EC_50_ of VGCV

A neutral red assay was used to quantify the inhibition of Ad cytopathic effect (CPE) by VGCV. Human A549 cells were plated in Dulbecco’s MEM containing 10% FBS at 8 × 10^3^ cells per well (96-well plates) 1 day prior to infection. Cells were infected with various Ad serotypes (9 wells per drug concentration) or mock-infected (drug only controls). Virus concentrations were as follows: Ad5 (1 PFU/cell), Ad6 (2 PFU/cell), or Ad4 (3 PFU/cell). At the end of the 1 h incubation, serially-diluted VGCV was added to the wells of the 96-well plate. Dilutions were set up as a 3-fold dilution series in serum-free DMEM. The final row of cells received medium with no drug added (the no drug control wells). Plates were incubated at 37 °C until the CPE reached ~50%–70% in the Ad-infected no drug treatment wells. At that time, 100 μL of neutral red (Sigma, St. Louis, MO, USA; diluted 1:10 in PBS) was added. After 1 h, plates were washed 3X with PBS. The neutral red was extracted by incubation with acidic alcohol solution (50% ethanol/1% acetic acid in water). The absorbance was read at 550 nm on a BioTek plate reader and data were analyzed using GraphPad Prism software.

To determine the 50% inhibitory concentration (IC_50_) for VGCV, A549 cells were set up as above one day prior to drug additions. VGCV was diluted in serial 1:3 dilutions (8 replicates per drug concentration) with no drug in the final row. Cell viability was assayed as above at 7 days post drug addition.

### 2.4. Immunofluorescent Staining and Western Blot

A549 cells, plated in 6-well plates on #1 glass cover glasses, were infected with representative Ad types, namely Ad5, Ad6 (species C), Ad7, Ad35 (species B), Ad4 (species E), Ad12 (species A), or Ad37 (species D) at 5 to 10 PFU/cell in serum-free DMEM. At 90 min p.i., the medium was replaced with DMEM containing 5% FBS and 500 μM VGCV or no drug.

For immunostaining, cells were fixed in 3.7% paraformaldehyde in PBS at 26 h p.i. and were permeabilized with cold methanol for 6 min and then rehydrated in PBS. Cells were stained with a rabbit anti-DBP antibody specific for the Ad-coded DNA Binding Protein (DBP) (gift of Maurice Green, Saint Louis University [24]) or a mouse monoclonal hexon-specific antibody reactive to all human Ads (2Hx-2, ATCC) [25]. Secondary antibodies were goat anti-rabbit IgG (Alexa Fluor 488 conjugate, Invitrogen Corp., Carlsbad, CA, USA) and goat anti-mouse IgG (Alexa Fluor 594 conjugate, Invitrogen Corp.). Cover glasses were mounted in mounting medium and images were taken with a Nikon DXM1200 digital camera mounted on a Nikon Optiphot microscope (Nikon, Melville, NY, USA) and using ACT-1 software (Nikon). All images were taken with the same exposure settings.

For Western blot, cells were infected and treated as described above. At 24 h p.i, the cells were harvested and 20 μg of protein from each sample was electrophoresed on 15% SDS polyacrylamide gels and electroblotted onto an Immobilon PVDF membrane (Millipore, Bedford, MA, USA). The membrane was treated with TBST (50 mM Tris-HCl (pH 7.6), 150 Mm NaCl, 0.2% Tween 20) containing 5% dry milk overnight at 4 °C, and then probed with a rabbit antiserum generated against a TrpE fusion protein containing amino acids 54–227 of the Ad2 protein pVIII (pVIII is an Ad capsid protein that is synthesized exclusively at late stages of infection), or the same rabbit antiserum used in immunofluorescent staining for DBP. Primary antibodies were incubated with the membranes in TBST containing 5% dry milk at room temperature. The secondary antibody was goat anti-rabbit IgG-HRP conjugate (Cappel, Durham, NC, USA). The blots were visualized using LumiGlo^®^ substrate (KPL, Gaithersburg, MD, USA).

### 2.5. Quantitative PCR (qPCR) Assay for Quantification of Ad5 Genomic DNA

We established a qPCR assay that allows direct quantitation of viral DNA copies in Ad5-infected A549 cells. The extraction procedure effectively releases viral DNA from infected cells and a 1/10 dilution of the extracts was sufficient to eliminate the impact of any PCR inhibiting factors present in these extracts. A549 cells seeded in 24-well plates at 1 × 10^5^ cells/well were either infected with Ad5 at 5 PFU/cell or mock-infected. After 90 min at 37 °C, the medium was aspirated and fresh DMEM containing serial dilutions (final concentrations ranging from 1 μM to 10 mM) of VGCV were added to the cells. Treatments were done in triplicate, and Ad5-infected cells with no-VGCV treatment as well as mock-infected cells receiving only VGCV were included as controls. At 24 h and 48 h p.i., the wells were washed twice with PBS, and the cells were lysed in 200 μL of lysis buffer (10 mM Tris-HCl (pH 8.0), 150 mM NaCl, 0.1% SDS, 0.5% NP40, 0.5% Tween-20, 0.5 mg/mL proteinase K) at 56 °C for 4–5 h. Proteinase K was then inactivated by boiling the reaction for 15 min. The samples were centrifuged at maximum speed in a microcentrifuge for 10 min, and 5 μL of 10-fold diluted supernatant was used as template in a 25 μL qPCR reaction. TaqMan-based qPCR was used to quantify viral genome copies. The reaction conditions were described previously [16]. Amplification was performed in duplicate for each sample using an ABI model 7500 genetic analyzer (Life Technologies, Grand Island, NY, USA). A standard curve generated using 10^2^ to 10^7^ copies of purified Ad5 viral genomic DNA was employed to determine absolute viral genome copy number per cell.

## 3. Results

### 3.1. VGCV Protects Immunosuppressed Hamsters Challenged Intravenously with Ad5, Even When Administered Starting from Four Days after Ad5 Challenge

**Figure 1:**
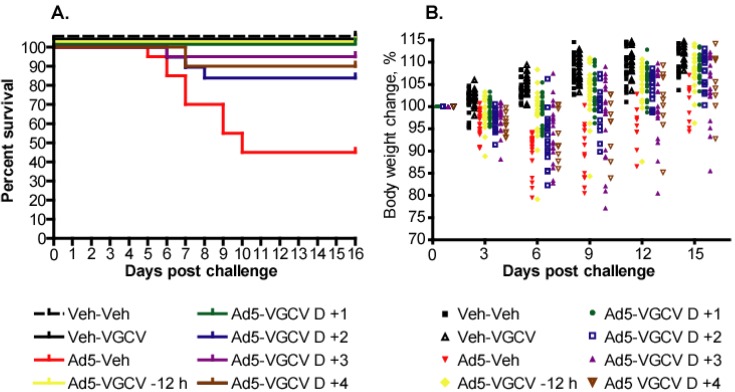
Valganciclovir (VGCV) administered therapeutically increased survival and reduced weight loss after intravenous Ad5 challenge in immunosuppressed Syrian hamsters. (**A**) Survival. Ad5-Vehicle *vs.* Ad5 VGCV −12 h *p* = 0.0001, Ad5-Vehicle *vs.* Ad5 VGCV D +1 *p* = 0.0001, Ad5-Vehicle *vs.* Ad5 VGCV D +2 *p* = 0.0181, Ad5-Vehicle *vs.* Ad5 VGCV D +3 *p* = 0.0008, Ad5-Vehicle *vs.* Ad5 VGCV D +4 *p* = 0.0265 (Log rank); (**B**) Body weight changes. Each symbol represents values from individual animals. Every 3rd day shown for clarity. Ad5-Vehicle *vs.* Ad5-VGCV −12 h *p* < 0.0001, Ad5-Vehicle *vs.* Ad5-VGCV D +1 *p* < 0.0001, Ad5-Vehicle *vs.* Ad5-VGCV D +2 *p* < 0.0001, Ad5-Vehicle *vs.* Ad5-VGCV D +3 *p* < 0.0001, Ad5-Vehicle *vs.* Ad5-VGCV D +4 not calculated (two-way ANOVA).

In order to determine whether VGCV is active against Ad5 challenge when applied therapeutically (*i.e.*, after challenge), we used the highest dose from the MTD experiment (200 mg/kg twice daily (b.i.d.) (Figure S1)). The dose of 200 mg/kg b.i.d. was found to be efficacious against i.v. Ad5 infection in CP-treated Syrian hamsters when administered prophylactically (Figure S2). We started the treatment for different groups of Ad5-infected hamsters at 12 h before or 1, 2, 3, or 4 days after challenge and evaluated the efficacy of the drug. We performed two such experiments that were almost identical, with the exception that one of the studies did not have a day 4 start time group. Here we are showing the combined results of these two parallel experiments (Figure 1).

Ad5 challenge alone caused 55% mortality in this study; this was reduced to 0% in the −12 h and D +1 groups, and to 16%, 5%, and 10% in the D +2, D +3, and D +4 groups, respectively (Figure 1A). All of these reductions were statistically significant (*p* < 0.0265). All the animals that were sacrificed ahead of schedule exhibited clinical signs associated with Ad5 infection (rapid weight loss, unthrifty look, dehydration, *etc.*) before becoming moribund.

The Ad5-infected, vehicle-treated animals began losing weight starting from after virus challenge to *ca.* 6–9 days p.i.; the surviving hamsters then slowly started to gain weight but never reached the weight of the vehicle-challenged control animals (Figure 1B). VGCV administration resulted in significantly smaller (*p* < 0.0001) magnitude of weight loss for all groups that were treated with the drug, and by the conclusion of the study these animals had body weights similar to vehicle-challenged hamsters. No evidence of toxicity was observed with the drug-only control hamsters.

Eight out of 10 Ad5-injected, vehicle-treated hamsters that were necropsied at 5 days p.i. had pale, yellow-mottled livers (data not shown). No animal was found with such pathology in the Ad5-VGCV −12 h and Ad5-VGCV D +1 groups. Eight out of 10, 5 out of 10, and one out of 5 hamsters exhibited liver pathology in the Ad5-VGCV D +2, Ad5-VGCV D +3, and Ad5-VGCV D +4 groups, respectively. All moribund hamsters sacrificed before schedule presented pathology characteristic of Ad5 infection. At 16 days p.i., most animals had recovered from infection; 3 hamsters from the Ad5-infected, vehicle-treated group, one animal in each of the Ad5-VGCV D +1, Ad5-VGCV D +2, Ad5-VGCV D +4 groups, and 2 animals in the Ad5-VGCV D +3 group had observable liver pathology.

For the samples collected at 5 days post challenge, the histopathological analysis revealed moderate to marked multifocal hepatocellular necrosis with intranuclear inclusion bodies in the livers of Ad5-infected, vehicle-treated animals (composite score 3.4) (Table 1). Similar pathology was described for the animals that were sacrificed moribund.

**Table 1:** Therapeutic administration of VGCV reduces the liver pathology induced by intravenous Ad5 challenge in immunosuppressed hamsters

Group	Animals affected	Severity (average histopathology scores for hepatocellular necrosis (a representative lesion) are shown)	Distribution
Vehicle-Vehicle	0/5	N/A	N/A
Vehicle-VGCV	0/5	N/A	N/A
Ad5-Vehicle	5/5	3.4	Multifocal
Ad5-VGCV −12	4/5	1.5	Multifocal
Ad5-VGCV +1	5/5	1.8	Multifocal
Ad5-VGCV +2	5/5	2.4	Multifocal
Ad5-VGCV+3	4/5	1.75	Multifocal

Hepatocellular necrosis with intranuclear inclusion bodies was observed in the livers of the VGCV-treated animals as well; however, the severity of the pathology was minimal to mild (composite score 1.5) in the −12 h group, minimal to mild (composite score 1.8) in the D +1 group, minimal to moderate (composite score 2.4) in the D +2 group, and minimal to mild (composite score 1.75) in the D +3 group (Table 1).

For the samples collected at 16 days post challenge, surviving animals showed signs of recovery, manifest by the absence of hepatocellular necrosis, and minimal to moderate subacute periportal inflammation, occasionally with Kuppfer cell nodules and mineralization.

**Figure 2:**
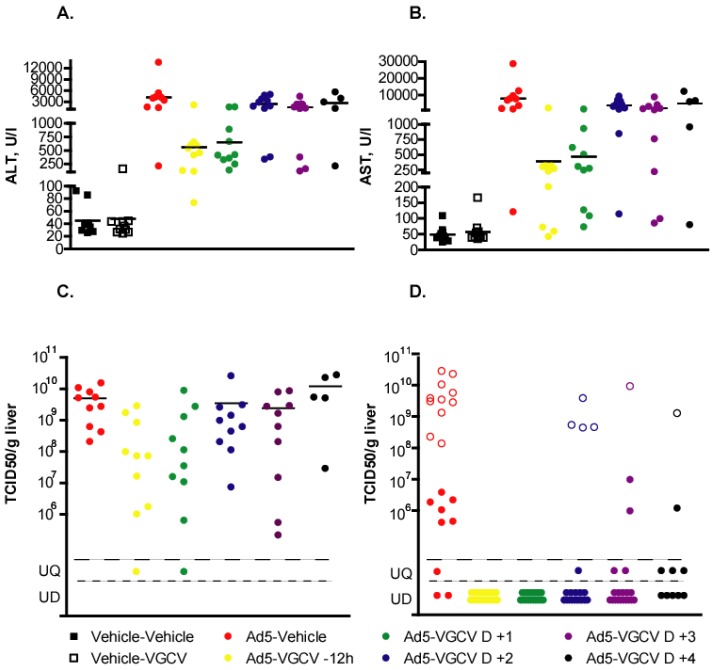
VGCV administered therapeutically significantly reduced the replication of Ad5 in the liver and consequent liver damage in immunosuppressed Syrian hamsters. Each symbol represents the value from an individual animal; the horizontal bar signifies the mean. Serum transaminase levels at 5 days p.i. (**A**) ALT: Ad5-Vehicle *vs.* Ad5-VGCV −12 h *p* = 0.0011, Ad5 *vs.* Ad5-VGCV D +1 *p* = 0.0029, Ad5 *vs.* Ad5-VGCV D +2 *p* = 0.2799, Ad5 *vs.* Ad5-VGCV D +3 *p* = 0.0355, Ad5 *vs.* Ad5-VGCV D +4 *p* = 0.4396; (**B**) AST: Ad5-Vehicle *vs.* Ad5-VGCV −12 h *p* = 0.0011, Ad5 *vs.* Ad5-VGCV D +1 *p* = 0.0007, Ad5 *vs.* Ad5-VGCV D +2 *p* = 0.1903, Ad5 *vs.* Ad5-VGCV D +3 *p* = 0.0355, Ad5 *vs.* Ad5-VGCV D +4 *p* = 0.3710 (Mann-Whitney U test); (**C**) Virus burden in the liver in samples collected at day 5. Ad5-Vehicle *vs.* Ad5-VGCV −12 h *p* = 0.0021, Ad5 *vs.* Ad5-VGCV D +1 *p* = 0.0089, Ad5 *vs.* Ad5-VGCV D +2 *p* = 0.1230, Ad5 *vs.* Ad5-VGCV D +3 *p* = 0.1655, Ad5 *vs.* Ad5-VGCV D +4 *p* = 0.8413 (Mann-Whitney U test); (**D**) Virus burden in the liver in samples collected at day 16. The empty symbols signify values from animals sacrificed moribund ahead of schedule. UD: undetectable, UQ: unquantifiable.

Serum was collected at necropsy and was analyzed for ALT and AST levels. As determined from the samples collected at 5 days p.i., VGCV treatment, even when started 1 day p.i., significantly reduced the magnitude of transaminase elevation caused by Ad5 replication (*p* = 0.0029 for ALT and *p* = 0.0007 for AST) (Figure 2A,B). At 15 days p.i., ALT and AST levels of all surviving animals were normal or only marginally elevated (data not shown).

At 5 days p.i., all VGCV-treated groups, with the exception of the group for which treatment started 4 days p.i., had lower virus burden in the liver (Figure 2C). The effect was most pronounced in the hamsters in the Ad5-VGCV −12 h (*p* = 0.0021) and Ad5-VGCV D +1 groups (*p* = 0.0089), for which an approximately 100-fold decrease in Ad5 titers was observed. At 16 days p.i., the virus burden was undetectable for most VGCV-treated hamsters, while most surviving hamsters in the Ad5-injected, vehicle-treated group still had high virus burden in the liver (Figure 2D).

### 3.2. VGCV Treatment Is also Effective in Male Syrian Hamsters

**Figure 3:**
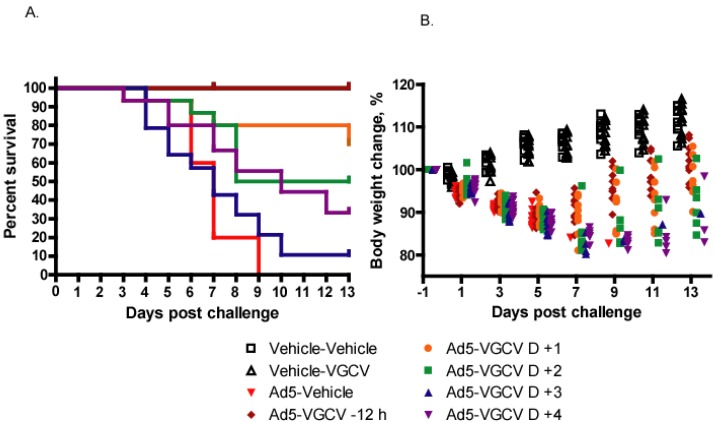
Therapeutic administration of VGCV reduces mortality and morbidity of Ad5-infected male Syrian hamsters. (**A**) Survival. Ad5-Vehicle *vs.* Ad5-VGCV −12 h *p* < 0.0001, Ad5-Vehicle *vs.* Ad5-VGCV D +1 *p* = 0.0005, Ad5-Vehicle *vs.* Ad5-VGCV D +2 *p* = 0.0018, Ad5-Vehicle *vs.* Ad5-VGCV D +3 *p* = 0.5264, Ad5-Vehicle *vs.* Ad5-VGCV D +4 *p* = 0.0152 (Log Rank test); (**B**) Body weight changes. Each symbol represents the value from an individual animal; data from every second day are shown for clarity’s sake. Significance was not computed.

In a follow-up experiment, we tested whether Ad5-challenged male hamsters responded differently to VGCV treatment than did females. In this study, we used the LD_90_ of Ad5 as challenge dose, which caused 100% mortality with the Ad5-infected, vehicle-treated group (Figure 3A). Notably, all prophylactically treated hamsters survived, and there was a significant improvement (*p* = 0.0018) of survival with the groups that were treated 1 or 2 days p.i., in which 70% and 55% of the hamsters survived, respectively (Figure 3A). In the first 5 days of the study, all Ad5-infected hamsters lost weight rapidly; however, animals that were treated with VGCV 1 day before or 1 day after virus challenge started recovering after Day 6–7 (Figure 3B).

For the purposes of gross pathology evaluation, animals sacrificed moribund before the Day 7 time point were grouped with animals that were scheduled to be sacrificed at that time. All 14 hamsters in the Ad5-Vehicle group had yellow, mottled, friable livers, as is characteristic for immunosuppressed hamsters infected with high doses of Ad5. None of the 5 hamsters presented such pathology in the Ad5-VGCV −12 h group, and only 3 out of 7 in both the Ad5-VGCV D +1 and Ad5-VGCV D +2 groups had liver lesions. In these latter groups, the animals with Ad-specific pathology were all sacrificed moribund ahead of schedule. The Ad5-VGCV D +3 and Ad5-VGCV D +4 groups had 9 out of 11 and 5 out of 9 hamsters with grossly abnormal livers, respectively. No significant findings were noted for the uninfected, vehicle- or VGCV-treated hamsters necropsied at 7 days post challenge.

The animals that were sacrificed moribund at various time points after the Day 7 scheduled sacrifice presented pathology characteristic of systemic Ad5 infection. One observation stands out as heretofore not seen with female hamsters in our model: we noticed that several of these Ad-infected animals presented with gross evidence of intraperitoneal bleeding.

At the conclusion of the study (Day 13), necrotic foci were observed in the liver of the hamsters in the Ad5-VGCV Day+2, +3, and +4 groups. The size and number of these lesions increased from occasional pinhead-sized foci in the Ad5-VGCV D +2 group to numerous 2–3 mm diameter foci in the Ad5-VGCV D +4 group. No gross pathology was observed with the hamsters in the Vehicle-Vehicle, Vehicle-VGCV, Ad5-VGCV −12 h, and Ad5-VGCV D +1 groups.

For the samples collected at 7 days post challenge, the histopathological analysis revealed moderate to marked multifocal hepatocellular necrosis with intranuclear inclusion bodies in the livers of Ad5-infected, vehicle-treated animals (composite score 3.7) (Table 2). Similar pathology was present in the animals that were sacrificed moribund.

Only 3 out of 5 and 4 out of 7 hamsters presented with hepatocellular necrosis with intranuclear inclusion bodies in the Ad5-VGCV -12 h and Ad5-VGCV D +1 groups, respectively, with a composite score of 0.6 (for both groups). Similar pathology was detected with the animals in the Ad5-VGCV D +2, Ad5-VGCV D +3, and Ad5-VGCV D +4 groups; however, the severity of the lesions was lower than in the case of Ad5-infected, vehicle-treated animals (Table 2). Surviving animals sacrificed at the conclusion of the study showed signs of recovery similar to those described for the experiment in which female hamsters were treated therapeutically with VGCV.

**Table 2:** Therapeutic administration of VGCV reduces the liver pathology induced by intravenous Ad5 challenge in immunosuppressed hamsters (The table shows pathology for animals sacrificed between 4 and 9 days p.i.)

Group	Animals Affected	Severity (average histopathology scores for hepatocellular necrosis (a representative lesion) are shown)	Distribution
Vehicle-Vehicle	0/5	N/A	N/A
Vehicle-VGCV	0/5	N/A	N/A
Ad5-Vehicle	15/15	3.7	Multifocal
Ad5-VGCV −12	3/5	0.6	Focal/Multifocal
Ad5-VGCV +1	4/7	0.6	Multifocal
Ad5-VGCV +2	10/11	2.1	Multifocal
Ad5-VGCV+3	13/14	2.9	Multifocal
Ad5-VGCV+4	9/10	3.1	Multifocal

**Figure 4:**
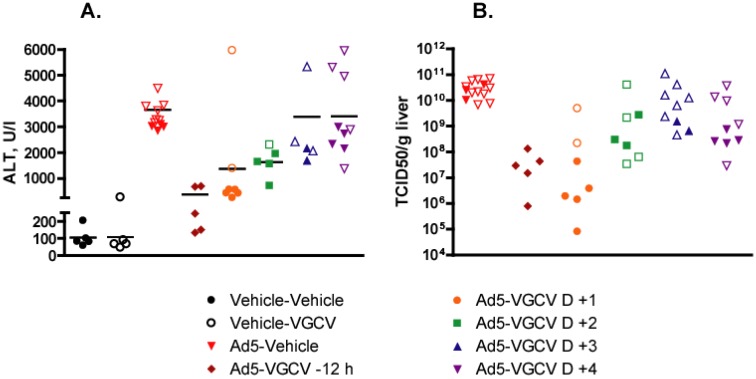
At 7 days post challenge, VGCV reduces the replication of Ad5 in the liver and mitigates Ad5-induced liver damage in male Syrian hamsters. Each symbol represents the value from an individual animal; the horizontal bar signifies the mean. Data from Ad5-infected animals sacrificed moribund ahead of schedule were included with the Day 7 time point; these values are denoted by empty symbols. (**A**) Serum ALT levels. Ad5-Vehicle *vs.* Ad5-VGCV −12 h *p* = 0.0019, Ad5-Vehicle *vs.* Ad5-VGCV D +1 *p* = 0.0100, Ad5-Vehicle *vs.* Ad5-VGCV D +2 *p* = 0.0019, Ad5-Vehicle *vs.* Ad5-VGCV D +3 *p* = 0.2418, Ad5-Vehicle *vs.* Ad5-VGCV D +4 *p* = 0.1887 (Mann-Whitney U-test); (**B**) Liver virus burden. Ad5-Vehicle *vs.* Ad5-VGCV -12 h *p* = 0.0016, Ad5-Vehicle *vs.* Ad5-VGCV D +1 *p* = 0.0004, Ad5-Vehicle *vs.* Ad5-VGCV D +2 *p* = 0.0044, Ad5-Vehicle *vs.* Ad5-VGCV D +3 *p* = 0.0489, Ad5-Vehicle *vs.* Ad5-VGCV D +4 *p* = 0.0033 (Mann-Whitney *U*-test).

Serum collected at necropsy was evaluated for ALT and AST (AST not shown) levels. For the purposes of evaluation, animals sacrificed moribund before the Day 7 time point will be grouped with animals that were scheduled to be sacrificed at that time. Consistent with the liver pathology observed at necropsy, animals in the Ad5-Vehicle group had highly elevated ALT levels (Figure 4A). VGCV treatment significantly reduced this elevation, even when administration of the drug started two days after Ad5 challenge (*p* = 0.0019). Notably, only hamsters that were sacrificed moribund ahead of schedule had highly elevated ALT levels in the Ad5-VGCV D +1 group (Figure 4A).

Infectious virus load in the liver of Ad5-infected animals was determined by TCID_50_ assay. At 7 days p.i., animals treated with VGCV had significantly lower virus burden in the liver, irrespective of when the treatment started (Figure 4B). The effect was most pronounced in the hamsters in the Ad5-VGCV −12 h and Ad5-VGCV D +1 groups, for which over 100-fold decrease in Ad5 titers was observed. Similar to the serum ALT levels discussed above, only animals sacrificed moribund had grossly high virus burden in the Ad5-VGCV D +1 group.

### 3.3. VGCV Inhibits the Growth of Human Ads *in Vitro*

**Figure 5:**
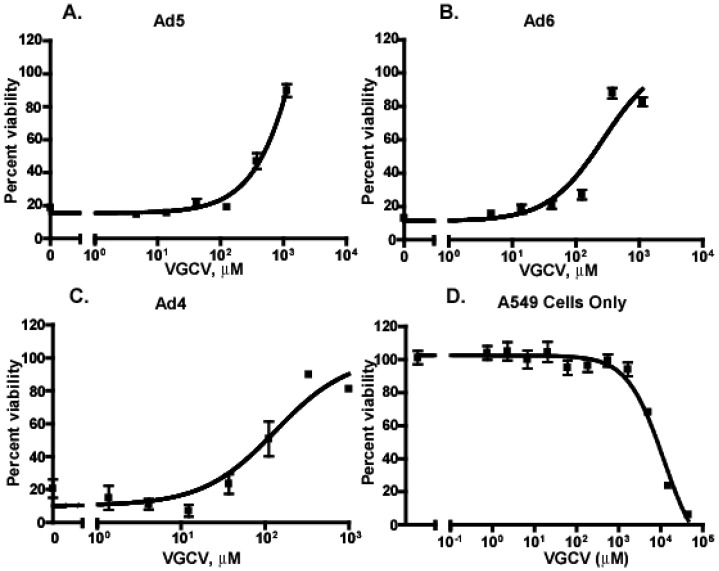
VGCV inhibits the growth of human Ads *in vitro*. A549 cells were infected with Ad5 (1 PFU/cell) (**A**), Ad6 (2 PFU/cell) (**B**), or Ad4 (3 PFU/cell) (**C**) and treated with serial dilutions of VGC*VS.* Cell viability was determined by neutral red assay at 5 d p.i. (Ad5, Ad6) or 7 d p.i. (Ad4). The IC_50_ for VGCV (**D**) was determined in parallel with neutral red addition at 7 d post drug addition.

An *in vitro* viability assay was used to determine the ability of VGCV to inhibit Ad infection in the human A549 cell line. In addition to Ad5, infections were done with Ad6 (Species C) and Ad4 (Species E). The assay was done at a multiplicity of infection (MOI) that would ensure that the majority of cells were infected (ranging from 1–3 PFU/cell). Serial dilutions of VGCV were added 1 h after addition of the virus (VGCV was not replenished over the course of the assay). At 5–7 days p.i., EC_50_ values ranged from 120.5 μM (Ad4) to 244.4 μM (Ad6) (Figure 5). Therefore, VGCV was able to inhibit multiple Ad serotypes *in vitro*. Inhibition of Ad7 and Ad35 (both Species B) also occurred with similar EC_50_ values (data not shown). A parallel plate was used to determine the IC_50_ for VGCV on A549 cells. At 7 days p.i., the IC_50_ was calculated to be 11.74 mM.

### 3.4. Treatment with VGCV Prevents Progression of Adenovirus Infection into the Late Stage

**Figure 6:**
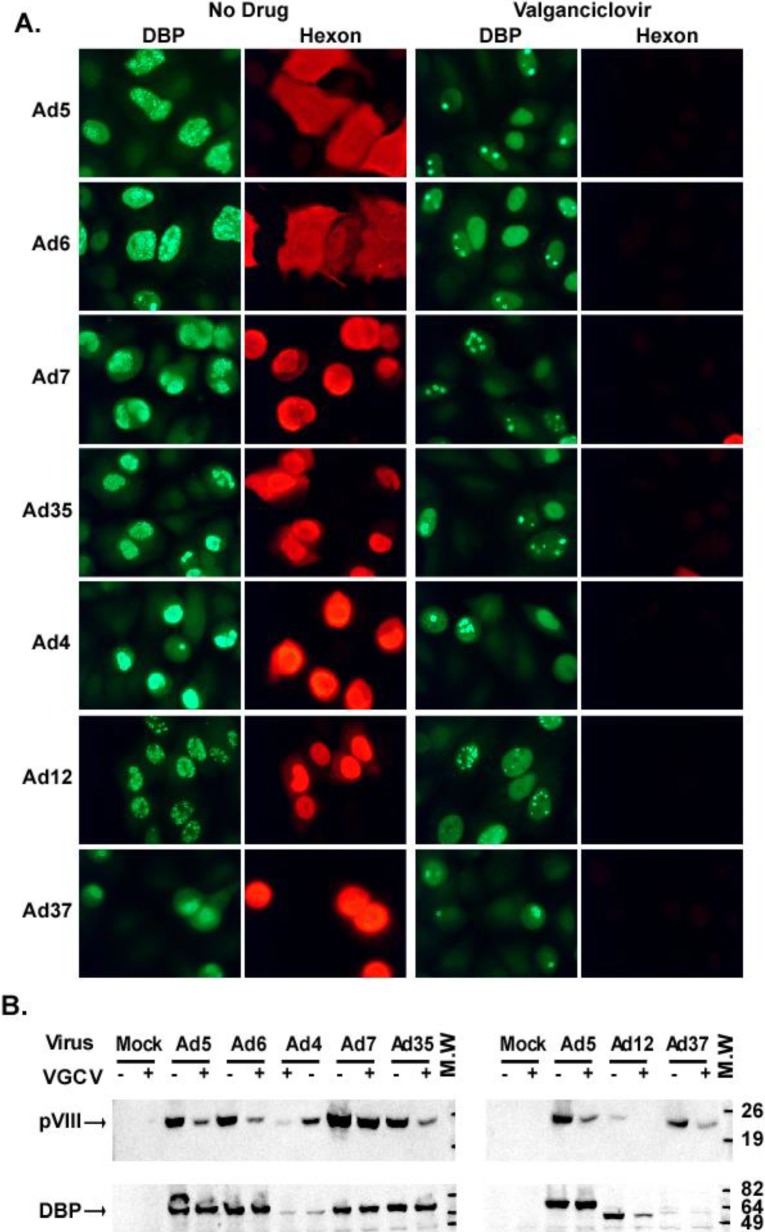
VGCV prevents adenoviral infection from advancing into the late phase. (**A**) Immunofluorescent staining for an early (DBP) and a late (hexon) Ad protein in VGCV-treated or untreated A549 cells, infected with species C (Ad5, 6), B (Ad7, 35), E (Ad4), A (Ad12), or D (Ad37) Ads. Uninfected cells did not stain with either antibody (data not shown); (**B**) Immunoblot staining for DBP and pVIII (another late Ad protein) in cells infected with the same types of Ads as in (A).

Ad infection can be divided into early and late phases; the latter is characterized by the expression of Ad late (mostly virion, e.g., the hexon protein) proteins and requires the initiation of synthesis of Ad genomic DNA [26]. We performed immunoassays to find out if VGCV-treatment inhibited the production of Ad late proteins. Expression of the Ad hexon protein was dramatically decreased in VGCV-treated A549 cells infected with Ads belonging to five different species (Figure 6A). To demonstrate that VGCV had no effect on processes upstream of viral DNA replication, we stained the same cells for DBP as well. DBP is an Ad protein that is synthesized initially prior to Ad DNA replication and whose synthesis continues well into the late stage of infection. In cells untreated with VGCV, DBP localized in a large number of replication centers; in some cases the entire nucleus stained positively for DBP (Figure 6A). This multitude of large replication centers, sometimes running together, which are seen at late stages of infection, are the site for Ad DNA replication and late gene transcription. In VGCV-treated cells, DBP was mostly confined to the original distinct replication centers associated with the input virus (this is an early DBP localization pattern; one infecting virion is thought to give rise to a single replication center) or showed uniform diffuse nuclear staining (a pattern seen in very early infection) (Figure 6A) [27,28], indicating that VGCV prevents the progression of the viral infection into the late phase.

These results were supported by an immunoblot, in which we showed that VGCV treatment greatly reduced the expression of protein pVIII, another Ad capsid protein, in Ad-infected cultures (Figure 6B). Conversely, no changes were seen in the expression of DBP (except for the Ad12 infection) (Figure 6B).

Taken together, these results indicate that the obstruction to Ad replication by VGCV takes place after early events of Ad infection (*i.e.*, receptor binding, uptake, escape from the endosome, transport to the nucleus, and early gene expression), and before the expression of Ad late proteins such as hexon and pVIII. Further, they point to the broad spectrum of inhibition by VGCV, inasmuch as seven different types from five Ad species reacted similarly to the drug.

### 3.5. VGCV Inhibits Ad5 DNA Replication

**Figure 7:**
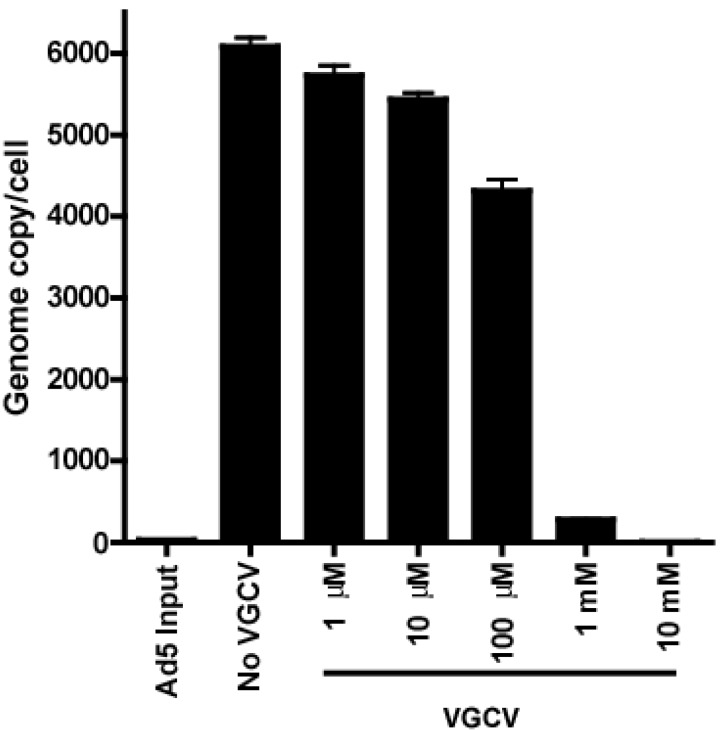
VGCV inhibits adenoviral DNA replication *in vitro*.

Figure 7 shows the inhibition by VGCV of Ad5 DNA replication. The number of Ad5 DNA copies per cell at 24 h p.i. reached approximately 6000 copies/cell in untreated cells. VGCV treatment at 1 μM did not show substantial inhibition of Ad5 DNA replication. In contrast, VGCV treatment at 100 μM significantly reduced viral DNA replication to 70% of the level in the untreated control, and VGCV treatment at 1 mM knocked down viral DNA replication by 25-fold. The same fold inhibition was observed at 48 h p.i. even though the viral DNA copies increased by nearly 100-fold at 48 h p.i. compared to that 24 h p.i. (data not shown). A dose response for VGCV is apparent in Figure 7. The calculated EC_50_ for VGCV to inhibit Ad5 DNA replication is 228 μM, which is in line with the EC_50_ in cell culture assays. Taken together, qPCR analysis of viral genomic DNA demonstrated potent anti-Ad5 activity of VGCV *in vitro*.

A549 cells were infected with Ad5 at 5 PFU/cell. The infection was treated with different concentrations of VGCV for 24 h. The viral DNA was quantified by qPCR; it is presented as viral genome copy number per cell. The means of three biological replicates ± standard deviation are shown. Ad5 *vs.* Ad5 + VGCV 1 μM *p* = 0.0649, Ad5 *vs.* Ad5 + VGCV 10 μM *p* < 0.01, Ad5 *vs.* Ad5 + VGCV 100 μM *p* < 0.01, Ad5 *vs.* Ad5 + VGCV 1 mM *p* < 0.01, Ad5 *vs.* Ad5 + VGCV 10 mM *p* < 0.01 (Mann-Whitney U-test).

### 3.6. VGCV Inhibits Ad5 Replication by Mechanisms other than that Described for Herpesviruses

We have shown elsewhere that ganciclovir (GCV), of which VGCV is a prodrug, is not phosphorylated in Ad5-infected cells, and that it is not incorporated into the viral genome [21]. Thus, VGCV (like GCV) probably inhibits Ad5 replication using alternative mechanisms. While the precise mechanism of this inhibition is unknown, here we present preliminary data suggesting two possible means of inhibition.

We investigated whether unphosphorylated VGCV can directly inhibit the Ad5 DNA polymerase (Ad5 Pol) *in vitro*. As shown in Figure S3A, purified Ad5 Pol enzyme can utilize dNTPs in a primer extension assay *in vitro* and addition of increasing concentrations of VGCV to the reaction mixture reduced the extension efficacy of Ad5 Pol. This suggests that direct inhibition of the Ad DNA polymerase might be one of the mechanisms by which VGCV inhibits Ad5 replication.

In searching for another mechanism of action for VGCV, we investigated whether a disturbance in nucleotide supply may be responsible for the inhibition of Ad replication. Optimal viral DNA synthesis requires an adequate amount of each nucleotide and appropriately balanced intracellular rNTP/dNTP pools. An imbalance of nucleotide pools could present a challenge for viral DNA and RNA polymerases to select their proper substrate. To test whether alteration of nucleotide pools may contribute to the anti-Ad activity of VGCV, qPCR was employed to quantify viral DNA replication following supplementing Ad5-infected, VGCV-treated cells with various nucleotides. In the study, Ad5-infected A549 cells were treated with 500 μM VGCV, and then the same concentration of each individual nucleotide or nucleotides in combinations were added to the infection culture. Viral genomic DNA was quantified 24 h p.i. As shown in Figure S3B, VGCV treatment reduced viral DNA replication approximately 6-fold compared to the untreated control. Exogenous addition of purine nucleotides did not restore viral replication; in fact, dGTP further repressed viral DNA replication compared to GCV treatment alone (Figure S3B). Interestingly, supplementing VGCV-treated, Ad5-infected cells with pyrimidine nucleotides (dCTP + dTTP) increased Ad5 DNA replication two-fold compared to VGCV alone-treated infections (Figure S3B); however, no further enhancement beyond the two-fold increase in Ad5 DNA replication was achieved with addition of three deoxynucleotides (dATP, dCTP, and dTTP) or all four nucleotides (+dNTPs). These data demonstrate that supplementation with pyrimidine nucleotides partially reversed VGCV’s inhibition of Ad5 DNA replication. This suggests that an intracellular imbalance of purine/pyrimidine nucleotides caused by VGCV treatment may be one of the mechanisms by which VGCV inhibits Ad5 replication.

## 4. Discussion

We have employed the permissive immunosuppressed Syrian hamster model to study the ability of VGCV to inhibit Ad5 replication in the liver and Ad5-induced pathology. This model provides an excellent approximation of disseminated Ad5 infection in humans. We found that VGCV has strong activity against Ad5, significantly reducing Ad5-induced mortality, weight loss, replication in the liver, and pathology in the liver.

We tested VGCV in a therapeutic setting, using a dose of 200 mg/kg/day, and administering the drug at 12 h prior to infection and Days +1, +2, +3, and +4 p.i. We found that VGCV significantly reduced Ad5 mortality, weight loss, and liver pathology at all time points of VGCV addition. At 5 days p.i., serum transaminase levels and the virus load in the liver were significantly reduced. Importantly, while 6 out of the 9 surviving Ad5-infected, untreated hamsters had high virus burden in the liver at the conclusion of the study, the majority of VGCV-treated animals were virus-free by this time. The same dose of therapeutically administered VGCV significantly reduced mortality and morbidity of male hamsters infected with the LD_90_ of Ad5. With animals that started receiving the treatment at the −12 h, D +1, and D +2 time points, virus burden in the liver was 100-fold lower than in the Ad5-Vehicle group at 7 days p.i.. Considering that virus titers in the liver become extremely high at even 1 to 3 days p.i. and that there is on-going virus replication [10,16,29], it is quite remarkable that VGCV was efficacious when treatment was initiated at 1 or 2 days p.i., and even at 3 or 4 days p.i. in increasing survival.

While further studies are needed to characterize the pharmacokinetics of VGCV in the hamster model, our results suggest that VGCV may be explored for possible use against disseminated Ad infections in immunocompromised patients. We have shown previously that GCV, the parental drug of VGCV, was active against Ad infection in hamsters, and there is anecdotal clinical evidence that GCV may be effective against Ad infection and pathogenesis in transplant patients [30,31,32,33,34,35]. In addition, treatment with 0.15% GCV ophthalmic gel improved the outcome of Ad conjunctivitis [36]. It seems reasonable that if GCV can be effective in the clinic, that VGCV should be effective as well. Previously, it was shown that GCV and VGCV treatments are equally efficacious against CMV viraemia in immunocompromised patients [37]. Currently, clinicians favor the orally available VGCV over GCV that has to be administered intravenously. The 200 mg/kg b.i.d. dose that was shown to be efficacious against disseminated Ad5 infection in hamsters is potentially clinically relevant, inasmuch as this corresponds to a calculated human equivalent dose of 27 mg/kg b.i.d., [38] and the recommended human dose of VGCV for the treatment of CMV retinitis is 15 mg/kg b.i.d. Additional studies are needed to determine the plasma concentration of the drug to establish the correlation between the dose used for the hamster studies and the human dose used in the clinic.

We have shown that the EC_50_ of VGCV against various types of Ads in A549 cells is between 120.5 μM (Ad4) and 244.4 μM (Ad6), while the IC_50_ is 11.7 mM. These values maybe overestimated, inasmuch as we do not know the rate of conversion of VGCV to GCV, the active compound, in this cell line. However, as the efficacy of esterases should equally influence the efficacy and toxicity of VGCV, the selectivity index (SI) value of 48 to 97 is probably valid.

Regarding the mechanism of action of VGCV, we showed that the drug inhibits Ad DNA replication (Figure 7) and the transition from the early to late stage of infection (Figure 6A,B); the latter event requires the initiation of Ad DNA replication. The question arises as to whether VGCV functions against Ad5 in a manner analogous to its effect on herpesviruses. With herpesviruses, GCV (generated from VGCV) must first be phosphorylated by a viral-coded thymidine kinase (herpes simplex virus) or a protein kinase (HCMV), and then GCV-monophosphate is further phosphorylated to GCV-triphosphate by cellular kinases. The GCV-triphosphate is incorporated into viral DNA where it acts as a DNA chain terminator (see Introduction). In a separate study in which we examined the efficacy of GCV against Ad5 in the immunosuppressed Syrian hamster model, we found that [^3^H]GCV was not phosphorylated (or was barely phosphorylated) in Ad5-infected cultured A549 or HepG2 cells and it was not incorporated into Ad5 DNA [21]. We could not conduct such a study with VGCV since radiolabeled VGCV is not available. It is also notable that Ads are not known to encode a kinase. While we cannot exclude the possibility that VGCV gets phosphorylated *in vivo*, we do not think that the mechanism of action of VGCV against Ad5 involves phosphorylation of VGCV or GCV. Interestingly, preliminary data indicates that VGCV inhibits Ad5 DNA replication in a cell-free assay (Figure S3A). Thus, direct inhibition of the Ad DNA polymerase may be part of the mechanism by which VGCV is effective against Ad5 replication in cell culture and in Syrian hamsters. We have also shown that supplementing Ad5-infected cells with pyrimidine nucleotides partially reversed the inhibition of viral DNA synthesis by VGCV, suggesting that disturbing the intracellular nucleotide balance may be another mechanism by which VGCV inhibits the replication of Ad5. We want to stress the preliminary nature of these data; clearly, more work is needed to clarify the mechanism of action of VGCV in this system.

## Supplementary Files

Supplementary File 1
