# Supplementary material for: Valganciclovir Inhibits Human Adenovirus Replication and Pathology in Permissive Immunosuppressed Female and Male Syrian Hamsters
Source: Viruses. 2015 Mar 23;7(3):1409–28. doi: 10.3390/v7031409 (PMC4379578; doi:10.3390/v7031409)
Supplement: Supplementary File 1 [file viruses-07-01409-s001.pdf]

Supplementary Data

1. Supplementary Methods

Determining the maximum tolerated dose (MTD) of VGCV in immunosuppressed Syrian hamsters. Five groups of animals were set up, consisting of 6 hamsters each. Hamsters in the first 4 groups were treated with the drug at 100, 125, 150, and 200 mg/kg/ b.i.d., respectively; animals in the control group received drug vehicle. The treatments were given orally (p.o.), twice daily (12 h apart), for 16 consecutive days. The animals were observed and weighed daily. The hamsters were sacrificed on day 16. Liver, kidney, lung, spleen, and intestinal tract were collected and subjected to histopathological and immunohistochemical analysis. Serum was analyzed for Na^+, K^+, Cl^-, Ca^{2+}, PO_4^{3-}, glucose, blood urea nitrogen, creatinine, total protein, albumin, globulin, cholesterol, triglyceride, alanine aminotransferase, aspartate aminotransferase, total bilirubin, alkaline phosphatase, and creatine kinase levels.

Determining the efficacy of prophylactically administered VGCV in Ad5-infected immunosuppressed hamsters. All hamsters were immunosuppressed using CP. CP was administered intraperitoneally at a dose of 140 mg/kg, and then twice weekly at a dose of 100 mg/kg. There were eight groups of animals, two uninfected and six Ad5 infected. There were 15 hamsters in each group. The uninfected (injected with virus vehicle only) groups were: (1) virus vehicle i.v. and drug vehicle p.o. and (2) virus vehicle i.v. plus 200 mg/kg valganciclovir p.o. twice daily (b.i.d). Animals in the infected groups received 2×10^{10} plaque forming units (PFU) of Ad5 i.v. There were six such groups, in which the animals received (1) virus (Ad5) i.v. and drug vehicle p.o.; (2) Ad5 i.v. plus 200 mg/kg valganciclovir p.o. b.i.d.; (3) Ad5 i.v. plus 100 mg/kg valganciclovir p.o. b.i.d.; (4) Ad5 i.v. plus 200 mg/kg valganciclovir p.o. once daily (q.d.); (5) Ad5 i.v. plus 50 mg/kg valganciclovir p.o. b.i.d.; (6) Ad5 i.v. plus 100 mg/kg valganciclovir p.o. q.d. VGCV was administered starting at day −1 and then given daily throughout the experiment.

The body weights and any signs of morbidity of the animals were recorded daily. At 5 days post challenge, 5 hamsters of each group (designated at the start of the experiment) were sacrificed, and gross pathological observation was performed. Serum and liver were collected. Virus burden in liver was determined by $TCID_{50}$ assay. The serum was analyzed for transaminase levels. The remaining 10 hamsters were sacrificed at 12 days post challenge. Thus, there were two endpoints for the study. One, collected from ten animals, was body weight gain/loss. The other, collected from five animals for the day-5 time point, was virus burden in the liver and serum transaminase levels.

Ad5 DNA Polymerase Primer Extension Analysis. The Ad5-coded DNA polymerase (Ad5 Pol) bearing a six-histidine affinity tag at the *C*-terminus was expressed from a baculovirus/Ad5 Pol vector in SF9 cells [40]. The Ad5 Pol was purified as described previously [40]. A substrate containing a 5' ^{32}P-labeled 20-m annealed to a template 30-m was used to test polymerase extension [40,41]. Twenty five nanomoles of radiolabeled substrate was used in a 20 μL of reaction buffer containing Ad5 Pol in 50 mM Tris-HCl (pH 7.5), 1 mg/mL bovine serum albumin (BSA), 5 mM $MgCl_2$, 1 mM dithiothreitol (DTT), 60 μM dNTPs, and 5% glycerol. Vehicle or VGCV (15, 25, 50, 100, 250, or 500 μM) was added to the reaction, and then the mix was incubated at 37 °C for 10 min. To stop the reactions, 20 μL of 2X termination dye (90% formamide (v/v), 10 mM EDTA, 0.01% bromophenol blue, and 0.01% xylene cyanole) was added, followed by heating for 5 min at 95 °C. Reaction products were electrophoresed on a 22.5%, 7 M urea polyacrylamide gel for 1 h and 30 min at 80 watts. The bands were visualized using a GE Healthcare PhosphorImager and analyzed using ImageQuant version 1.2 software. A representative gel of an experiment performed in triplicate is shown in the figure.

Restoring the balance of nucleotide pools after treatment with GCV. A549 cells seeded in 24-well plates at 1 × 10^5 cells per well were mock-infected or infected with Ad5 at 5 PFU/cell. After 1.5 h at 37 °C, the infection media was aspirated and replaced by DMEM containing 500 µM of VGCV, and then 500 µM of dNTPs were added to the culture either individually or in combination. Each treatment was done in triplicate, and Ad5-infected cultures receiving no VGCV and Ad5-infected cultures receiving VGCV but no nucleotides were included as controls in each plate. At 24 h p.i., DNA was extracted and qPCR was performed as described in above to quantify the Ad genome copy number.

2. Supplementary Results

For immunosuppressed Syrian hamsters, the oral MTD of VGCV is higher than 200 mg/kg twice daily (b.i.d.). Immunosuppressed hamsters were administered up to 200 mg/kg VGCV b.i.d. (see Methods). None of the tested doses caused a slower rate of body weight gain than observed with animals treated with vehicle only (Figure S1). Further, no aberrant serum chemistry parameters or histopathology observations were noted for any of the dose levels (data not shown).

Prophylactically administered VGCV mitigates the pathogenicity of Ad5 in Immunosuppressed Syrian hamsters. VGCV reduced the extent of body weight loss (Figure S2A), reduced the level of alanine aminotransferase (ALT) in the serum (this reached statistical significance only for the 200 mg/kg b.i.d. dose) (Figure S2B), and reduced virus burden in the liver (Figure S2C). There is an observable dose effect, with the highest dose (200 mg/kg b.i.d.) practically abolishing the majority of pathology, and even the two lowest doses (50 mg/kg b.i.d. and 100 mg/kg q.d.) having a beneficial effect.

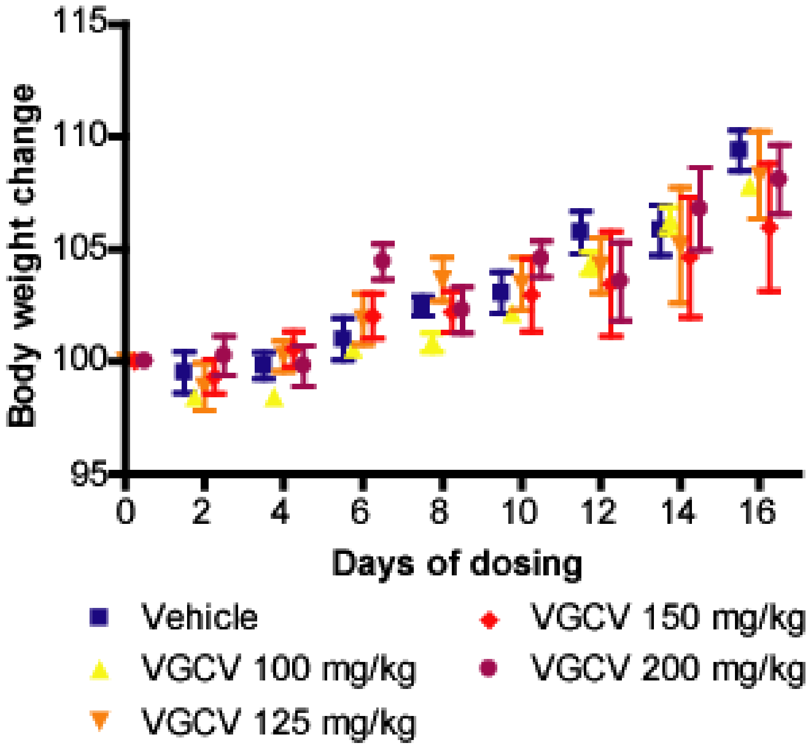

Figure S1. The MTD of VGCV in immunosuppressed hamsters is higher than 200 mg/kg b.i.d. The indicated doses were given p.o. twice daily for the duration of the study. The symbols represent the group mean. Whiskers signify the SEM.

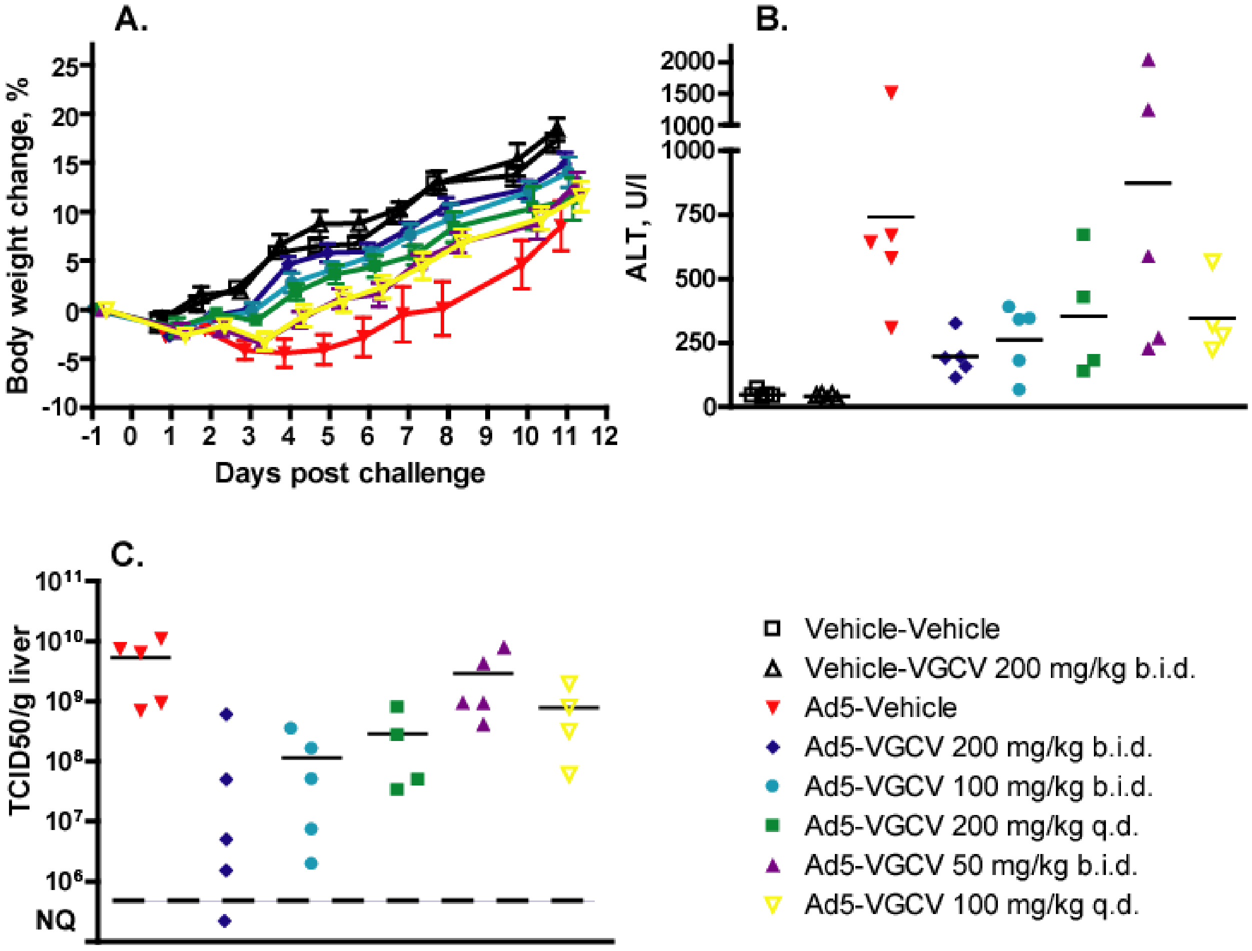

Figure S2. Prophylactically administered VGCV mitigates the pathogenicity of Ad5 in Immunosuppressed Syrian hamsters. (**A**) Body weight changes. The symbols represent the group mean. Whiskers signify the standard error of the mean. Ad5-Vehicle *vs.* all Ad5-VGCV groups $p < 0.0001$ (two-way ANOVA); (**B**) Serum ALT levels. Ad5-Vehicle *vs.* Ad5-VGCV 200 mg/kg b.i.d. $p = 0.0159$; Ad5-Vehicle *vs.* Ad5-VGCV at all other dose levels $p > 0.05$ (Mann-Whitney U-test); **C**. Infectious virus load in the liver. Ad5-Vehicle *vs.* Ad5-VGCV 200 mg/kg b.i.d. $p = 0.0079$; Ad5-Vehicle *vs.* Ad5-VGCV 100 mg/kg b.i.d. $p = 0.0079$; Ad5-Vehicle *vs.* Ad5-VGCV 200 mg/kg q.d. $p = 0.0317$; Ad5-Vehicle *vs.* Ad5-VGCV 50 mg/kg b.i.d. $p = 0.5476$; Ad5-Vehicle *vs.* Ad5-VGCV 100 mg/kg q.d. $p = 0.1111$ (Mann-Whitney U-test) For (**B**) and (**C**), each symbol represents data from an individual animal; the horizontal bar represents the standard error of the mean.

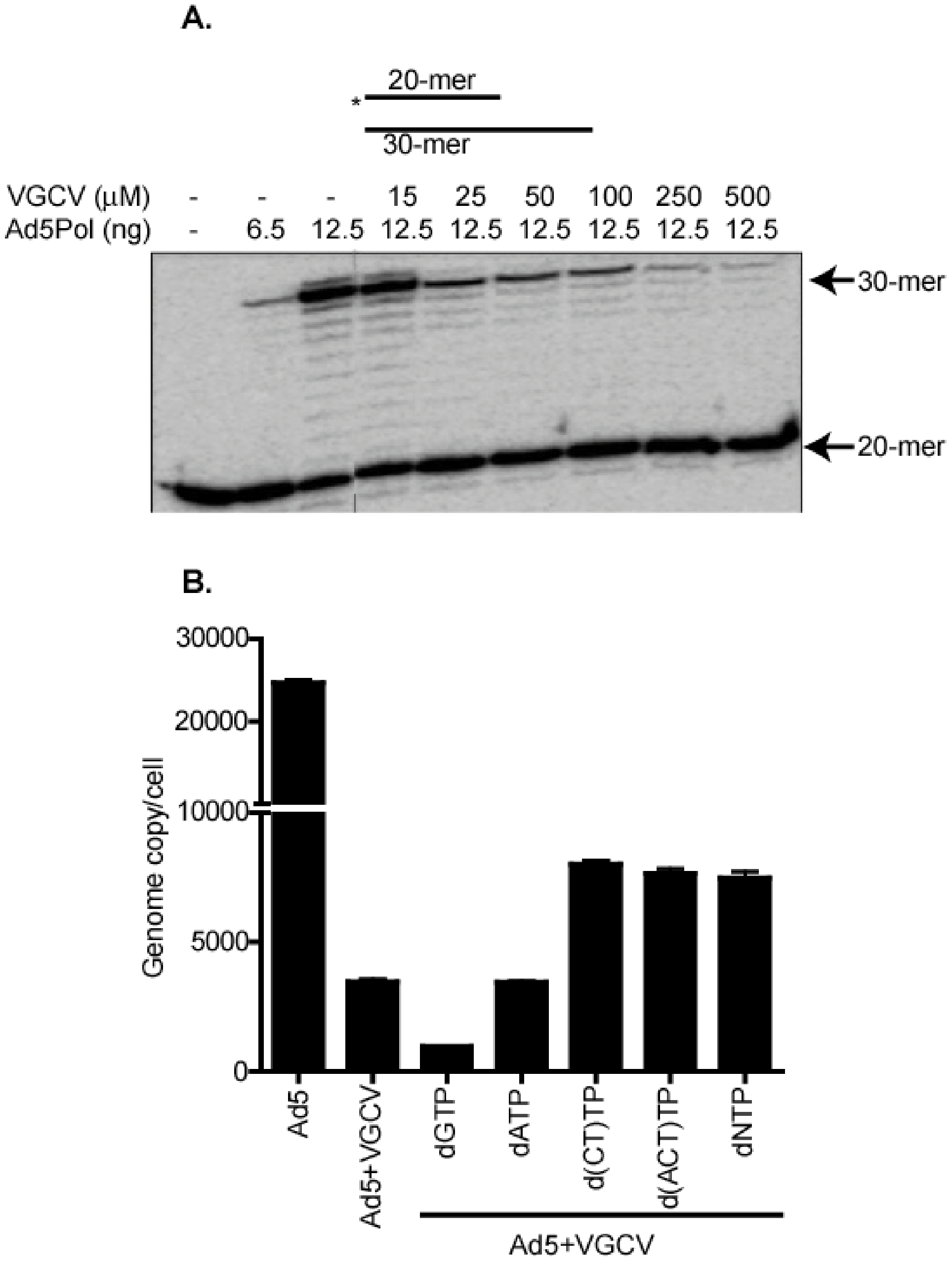

Figure S3. VGCV may inhibit the replication of Ad5 using alternative mechanisms. (**A**) VGCV reduces Ad5 DNA polymerase extension efficiency. Nucleotide extension by Ad5 DNA polymerase was assayed on a substrate containing a 5' labeled 20-mer annealed to a 30-m template in the absence or presence of increasing amounts (15 to 500 μM) of VGCV. The substrate is depicted above the figure, with the asterisk denoting the location of the radiolabel. The 20-m labeled primer, the 30-m fully extended product and the intermediate extension products are noted in the figure; (**B**) Balancing nucleotide pools partially reverses VGCV's inhibition of Ad DNA replication. Ad5-infected A549 cells were treated with 500 μM VGCV, and the same concentration of each individual nucleotide or nucleotides in combinations were added to the infection culture. Viral genomic DNA was extracted 24 h p.i. and quantified by qPCR. Viral DNA copy number per cell is presented in the graph. The Ad5 + GCV group was significantly different ($p < 0.01$, Mann-Whitney U-test) from all groups supplemented with nucleotide(s) except dATP.
